# A novel metric of reliability in pressure pain threshold measurement

**DOI:** 10.1038/s41598-021-86344-6

**Published:** 2021-03-25

**Authors:** Bernard Liew, Ho Yin Lee, David Rügamer, Alessandro Marco De Nunzio, Nicola R. Heneghan, Deborah Falla, David W. Evans

**Affiliations:** 1 School of Sport, Rehabilitation and Exercise Sciences, University of Essex, Essex, UK; 2 Centre of Precision Rehabilitation for Spinal Pain, School of Sport, Exercise and Rehabilitation Sciences, University of Birmingham, Edgbaston, Birmingham, B15 2TT UK; 3 Department of Statistics, Ludwig-Maximilians-Universität München, Munich, Germany; 4 LUNEX International University of Health, Exercise and Sports, Differdange, Luxembourg; 5 Research Centre, University College of Osteopathy, London, UK

**Keywords:** Physical examination, Statistics

## Abstract

The inter-session Intraclass Correlation Coefficient (ICC) is a commonly investigated and clinically important metric of reliability for pressure pain threshold (PPT) measurement. However, current investigations do not account for inter-repetition variability when calculating inter-session ICC, even though a PPT measurement taken at different sessions must also imply different repetitions. The primary aim was to evaluate and report a novel metric of reliability in PPT measurement: the inter-session-repetition ICC. One rater recorded ten repetitions of PPT measurement over the lumbar region bilaterally at two sessions in twenty healthy adults using a pressure algometer. Variance components were computed using linear mixed-models and used to construct ICCs; most notably inter-session ICC and inter-session-repetition ICC. At 70.1% of the total variance, the source of greatest variability was between subjects (${\sigma }_{subj}^{2}$ = 222.28 N^2^), whereas the source of least variability (1.5% total variance) was between sessions (${\sigma }_{sess}^{2}$ = 4.83 N^2^). Derived inter-session and inter-session-repetition ICCs were 0.88 (95%CI: 0.77 to 0.94) and 0.73 (95%CI: 0.53 to 0.84) respectively. Inter-session-repetition ICC provides a more conservative estimate of reliability than inter-session ICC, with the magnitude of difference being clinically meaningful. Quantifying individual sources of variability enables ICC construction to be reflective of individual testing protocols.

## Introduction

Assessing the sensitivity of body tissues in response to mechanical pressure is a fundamental element of the clinical examination for the patient with pain^1^. Pain thresholds are a commonly used measure within quantitative sensory testing (QST) paradigms; the pressure pain threshold (PPT) is the minimum quantity of pressure that induces a painful sensation when applied to a particular body site^2^. The most frequently employed method to measure a pain threshold involves continuously increasing the magnitude of stimulus (usually at a constant rate) until pain is evoked; this is known as the ascending method of limits^3^.

PPT measurement is typically repetitive in nature. It can be undertaken in multiple subjects^4,5^, by multiple assessors^5^, over multiple sessions^6,7^, at multiple body sites^4,8,9^, with multiple repetitions at each site^4,5,7,9,10^. This repetitive nature requires that sources of variability between measurements be identified and quantified.

Few studies have identified and quantified different sources of variability during PPT measurement^11,12^, with most reporting the relative ratio between variabilities: the Intraclass Correlation Coefficient (ICC)^13,14^. For example, for a PPT evaluation across different sessions and different subjects, the relevant inter-session ICC can be calculated using^15^:

1 $$ICC(session) = \frac{{\sigma }_{subj}^{2}}{{\sigma }_{subj}^{2} + {\sigma }_{sess}^{2}}$$

where ${\sigma }_{subj}^{2}$ represents the inter-subject variance and ${\sigma }_{sess}^{2}$ represents the inter-session variance. A high $ICC\left(session\right)$ could be due to a small ${\sigma }_{sess}^{2}$ or a large ${\sigma }_{subj}^{2}$, the latter ‘diluting’ variability from different sessions. Knowing the values of individual variabilities from which an ICC is constructed may have significant implications, because strategies that reduce between-session variation could be very different from those that reduce between-subject variation.

The traditional approach to formulating ICC, implemented within most statistical software applications (e.g. IBM SPSS Statistics), possesses several limitations with regards to the evaluation of PPT testing. Foremost, this traditional approach permits only two sources of variation (e.g. multiple sessions and subjects). Yet, as mentioned, PPT evaluations usually encompass more than two sources (e.g. subjects, sites, repetitions, sessions, assessors). As such, researchers are required to either collapse the data into two sources by averaging, before proceeding with their ICC calculation^5^, or instead perform multiple ICC calculations^16^.

Averaging has the disadvantage of omitting potentially important sources of variation. For example, a previous study reported an inter-session ICC of 0.70 calculated using traditional statistical software^11^. However, calculating ICC using individual sources of variance ($\frac{{\sigma }_{p}^{2}+ {\sigma }_{r}^{2}+ {\sigma }_{pr}^{2} }{{\sigma }_{total}^{2}})$^11^ produces a value of 0.66 instead. In addition, PPT measurements obtained from different sessions implies that they are obtained from different repetitions^17^. By not accounting for the natural variation associated with repetitions, the calculated inter-session ICC may therefore be overoptimistic.

Calculating multiple ICC values is also disadvantageous because a single ‘global’ estimate of PPT testing reliability cannot be derived. For example, one study reported ICC values ranging from 0.85 to 0.98 at different sites of the lumbar region^16^. Using established criteria^18^, these ICC values could have been interpreted as evidence of either good or excellent reliability for PPT measurement at the lumbar region^16^, which therefore remains ambiguous.

Given that few studies have reported values of different sources of measurement variability during PPT measurement, the primary purpose of the present study was to quantify and report those relevant to the present investigation. A secondary aim was to demonstrate how ICCs can be constructed from individual variance components. A third aim was to illustrate how the identification of individual sources of variability can help researchers and clinicians optimise the reliability of PPT measurement.

## Methods

### Participants

Healthy adults were recruited from the student population of a university in the UK. Inclusion criteria were: (1) no history of musculoskeletal pain requiring healthcare within the preceding 3 months, (2) no musculoskeletal pain at the time of testing and (3) ability to lie in a prone position for at least 30 min without discomfort. Exclusion criteria were: (1) inability to understand and follow instructions in verbal and written English, (2) any health condition potentially causing sensory deficits, such as diabetes mellitus or neurological disorders, (3) any history of chemotherapy, (4) currently taking medication that can affect sensation, and (5) currently pregnant. Participants were asked to limit intake of caffeine, alcohol and any medication that can cause sleepiness or analgesia for the 24-h prior to each testing session. The procedure was explained and written informed consent was obtained before data collection commenced. The study was approved by the ethics committee of the School of Sport, Exercise and Rehabilitation Sciences, University of Birmingham. All research was performed in accordance with the Declaration of Helsinki, and current guidelines and regulations were adhered to.

### Sample size

We calculated the sample size using the *ICC.Sample.Size* package in R software, which is based on Eq. (1)^19^. Given a null hypothesis ICC value of 0.8, the alternative hypothesis value of 0.9 and the number of sessions set to two, 18 participants were needed to achieve 80% power at a 5% significance level. Our recruitment target was therefore set at 20 participants to account for potential withdrawals.

### Study design

The study was a test–retest observational design with no experimental intervention. All testing procedures were performed within a dedicated sensory testing laboratory, in which temperature could be controlled at 22.0 ± 1.0 °C. For each participant, two testing sessions were performed by the same rater, with a minimum of 48 h^8^, and a maximum of 7 days (168 h) between sessions. The testing procedure within each session was the same.

### Equipment

**Figure 1:**
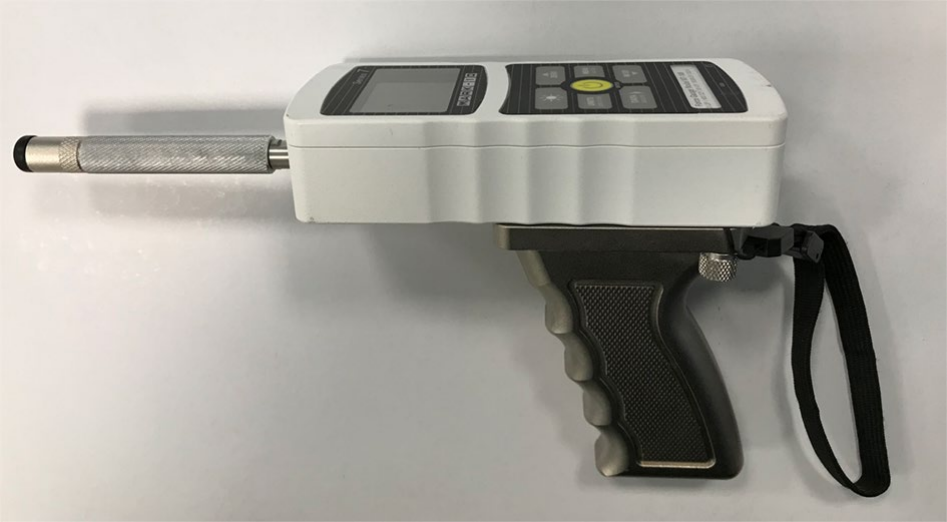
Digital algometer used to collect pressure pain threshold data.

PPT measurements were recorded using a configurable digital pressure algometer system^20^. This incorporated a laboratory-grade digital force gauge (Series 7, Mark-10, USA), fitted with a pistol grip and detachable hard rubber tip with contact area of 1.2 cm^2^ (Fig. 1). To ensure a constant and accurate rate of force application, the algometer was connected to a desktop computer with monitor via a 16-bit data acquisition board (NI USB-6001, National Instruments, USA). The computer ran a bespoke software application, developed using LabView software (National Instruments, USA), that provided visual real-time force feedback and guidance to direct the rater throughout testing. A safety limit guideline was set at 150 N, equivalent to 1000 kPa when used with the 1.2 cm^2^ contact tip. A handheld ‘trigger’ button was included in the system so that participants could provide instantaneous audible and visual responses to the rater; force values from these responses were automatically recorded by the software.

### Rater training

The rater was a postgraduate student with 3-years clinical experience as a physiotherapist, but minimal experience in PPT testing prior to the study. The rater was trained to use the algometer by supervising researchers with considerable experience in PPT testing and the apparatus. The correct technique for measuring PPT, with the contact tip of the algometer perpendicular to the skin and load increasing at a constant rate, was rehearsed before commencing participant testing in order to improve repeatability of force application^21^. The rater was trained to apply pressure at a constant and controlled loading rate with the use of the aforementioned LabView software application, which provided real-time visual feedback.

### Testing procedure

Participants completed a brief questionnaire, which included demographic data, health status, current medication intake and whether they were experiencing any pain at all. An explanation and demonstration of testing procedures were given to participants prior to testing; one practice PPT test on the forearm was provided to familiarise participants with the testing procedure and to ensure recognition of a painful pressure stimulus^22^. Participants were then asked to lie prone on a padded clinical plinth with a facial breathing hole (Akron, ArjoHuntleigh, UK), at which point the two testing sites (bilateral paraspinal regions at the level of L4/5, 2 cm from the midline)^5^ were marked by the rater with a semi-permanent surgical skin-marking pen (Schuco Ltd, UK). The order of site testing (right, left) was randomly allocated at each session using a computer software application (Random.org, Republic of Ireland). All verbal instructions were standardised during the test^23^. One series of ten consecutive PPT measurements were taken at each of the two testing sides, using a constant loading rate of 5 N/s, with an inter-stimulus interval of thirty seconds between repetitions^24,25^. This inter-stimulus interval was chosen to avoid the phenomenon of ‘wind up’, which is primarily due to the relatively long duration of excitatory synaptic potentials evoked from stimulated C-fibre nociceptors^26,27^. Participants were not given the opportunity to view the force–time readings displayed on the monitor. Data were automatically saved to the computer in pre-configured comma-separated variables files by the LabView software application.

### Statistical analysis

#### Quantifying sources of variability

To quantify variance components, we constructed a linear mixed effects model with the ‘lme4′ package for R statistical software. The following linear model was specified:

2 $${PPT}_{ijkl}=site + {subject}_{i} + {session}_{ij} + {side}_{ik} + {sss}_{ijk} + {repetition}_{l}$$

where ${PPT}_{ijkl}$ represents a PPT value of the *ith* subject, *jth* session, *kth* side, *lth* repetition; $site$ represents the mean (fixed effect) PPT value or ‘intercept’; ${subject}_{i} \sim N \left(0, {\sigma }_{subj}^{2}\right)$ represents the subject-specific random effect; ${session}_{ij }\sim N \left(0, {\sigma }_{sess}^{2}\right)$ represents the session-nested-within-subject random effect; ${side}_{ik }\sim N \left(0, {\sigma }_{side}^{2}\right)$ represents the side-nested-within-subject random effect; ${sss}_{ijk }\sim N \left((0, {\sigma }_{sss}^{2}\right)$ represents the session-side random interaction effect for each subject *i*; and ${repetition}_{l} \sim N \left(0, {\sigma }_{reps}^{2}\right)$ represents the residual term for the *l*th repetition. All unknown parameters were calculated using the residual maximum likelihood (REML) method.

#### Constructing the ICC

One advantage of quantifying individual sources of variability is that different ICC variants can be calculated, even for situations with a completely different setup to those from which they were derived^17^. In the present investigation, the inter-session ICC could be formulated as:

3 $$ICC \left(session\right)= Corr \left({PPT}_{ijkl}, {PPT}_{i{j}{^{\prime}}kl}\right)=\frac{{\sigma }_{subj}^{2} + {\sigma }_{side}^{2} + {\sigma }_{reps}^{2} }{{\sigma }_{subj}^{2} + {\sigma }_{sess }^{2} + {\sigma }_{side}^{2} + {\sigma }_{sss}^{2} + {\sigma }_{reps}^{2}}$$

where $Corr$ means correlation, ${PPT}_{ijkl}$ represents a PPT value of the *ith* subject, *jth* session, *kth* side, *lth* repetition, and ${PPT}_{i{j}{^{\prime}}{kl}{^{\prime}}}$ represents a PPT value of the same subject, side and repetition, measured at a different session. PPT measurements collected at different sessions must also imply they are obtained from different repetitions. Hence, the ICC value for inter-session-repetition should be considered a more comprehensive model of reliability, and can be quantified by:

4 $$ICC \left(session, reps\right)= Corr \left({PPT}_{ijkl}, {PPT}_{i{j}{^{\prime}}k{l}{^{\prime}}}\right)= \frac{{\sigma }_{subj}^{2} + {\sigma }_{side}^{2} }{{\sigma }_{subj}^{2} + {\sigma }_{sess }^{2} + {\sigma }_{side}^{2} + {\sigma }_{sss}^{2} + {\sigma }_{reps}^{2}}$$

where $Corr$ means correlation, ${PPT}_{ijkl}$ represents the PPT value of the *ith* subject, *jth* session, *kth* side, *lth* repetition, whereas ${PPT}_{i{j}{^{\prime}}k{l}{^{\prime}}}$ represents the PPT of the same subject, different session, same side and different repetition.

5 $$ICC \left(session, side, reps\right)= Corr \left({PPT}_{ijkl}, {PPT}_{i{j}{^{\prime}}{k}{^{\prime}}{l}{^{\prime}}}\right)= \frac{{\sigma }_{subj}^{2} }{{\sigma }_{subj}^{2} + {\sigma }_{sess }^{2} + {\sigma }_{side}^{2} + {\sigma }_{sss}^{2} + {\sigma }_{reps}^{2}}$$

where $Corr$ means correlation, ${PPT}_{ijkl}$ represents the PPT value of the *ith* subject, *jth* session, *kth* side, *lth* repetition, ${PPT}_{i{j}{^{\prime}}{k}{^{\prime}}{l}{^{\prime}}}$ represents the PPT of the same subject, different sessions, different sides and different repetitions.

#### Optimising PPT measurement

Another advantage of quantifying individual sources of variability is that these values can be used to design the setup most likely to increase the reliability of PPT measurement. For example, if subjects are being tested on two sessions, and each session involved testing on both sides, the variance components approach allows the assessor to determine the optimal number of repetitions (*L)* to ensure that the inter-session reliability crosses a given reliability threshold:

6 $$ICCk\left(session\right)= Corr \left(\stackrel{-}{{PPT}_{ij}}, \stackrel{-}{{PPT}_{i{j}{^{\prime}}}}\right)= \frac{{\sigma }_{subj}^{2} }{{\sigma }_{subj}^{2}+ {\sigma }_{sess }^{2}+ \frac{{\sigma }_{side}^{2}}{K}+ \frac{{\sigma }_{sss}^{2}}{K}+ \frac{{\sigma }_{reps}^{2}}{KL}}$$

where $Corr$ means correlation, $\stackrel{-}{{PPT}_{ij}}$ represents the average PPT value of the *i*^*th*^ subject, and *j*^*th*^ session, $\stackrel{-}{{PPT}_{i{j}{^{\prime}}}}$ represents the average PPT value of the same subject and different session; $K$ represents the number of sides from which PPT values are obtained, and *L* representing the number of repetitions over which to average the PPT values. To clarify, the ‘*k*’ in *ICCk* relates to standard ICC nomenclature^13^, and does not refer to the side (laterality) being tested. For $ICCk\left(sess\right)$, we varied *L* from *l* = 2, … , 10 repetitions and calculated the $ICCk \left(session\right)$, $ICC \left(session, reps\right)$, $ICC \left(session, side, reps\right)$, and $ICCk\left(session\right)$, using parametric bootstrapping with 1,000 iterations to derive 95% confidence intervals (CI).

#### Interpretation and reporting

The guidelines of Shrout^28^ were used to interpret ICC values: substantial reliability > 0.80; moderate reliability > 0.60 to 0.80; fair reliability > 0.40 to 0.60; slight reliability > 0.10 to 0.40; and, virtually no reliability < 0.10. Mean and standard deviations (SD) were calculated for all continuous variables of demographic data. PPT values are reported in newtons (N) and variance components of force data are reported in N^2^. All data, analysis codes, and results can be found on the following software repository: https://github.com/bernard-liew/2020_ICCvarComp.

## Results

**Figure 2:**
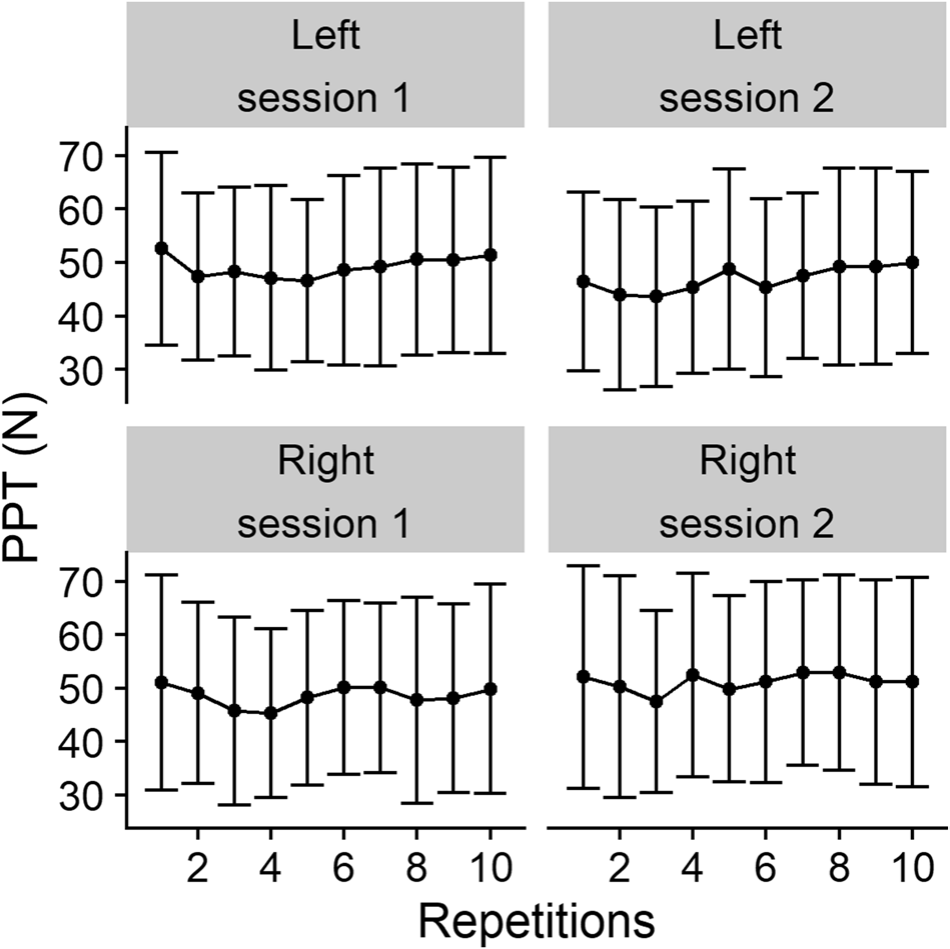
Group mean (error bars as standard deviation) of PPT values (N) for the lumbar paraspinal sides at each testing session.

We recruited 20 participants, the descriptive characteristics of whom can be found in Table 1. PPT values per repetition for each side, averaged across all subjects, are displayed for both sessions in Fig. 2.

**Table 1:** Summary of participant characteristics.

Number of participants	20
Male:Female	8:12
Age in years [Mean (SD)]	24.6 (2.4)
Inter-session hours [Median (Q1, Q3)]	48.5 (48.0, 74.0)
**Ethnicity**	
White	3
Asian-Chinese	12
Other Asian	4
Other	1

*SD* Standard deviation.

Q1 First quartile (25%) value.

Q3 Third quartile (75%) value.

At 70.1% of the total variance, the source with the greatest variation was ${\sigma }_{subj}^{2}$ = 222.28 (95% CI: 111.20 to 436.96) N^2^. This was followed by ${\sigma }_{reps}^{2}$ = 45.77(95% CI: 41.35 to 50.85) N^2^, which accounted for 14.4% of total variance; ${\sigma }_{sss}^{2}$ = 34.63 (95% CI: 17.77 to 60.86) N^2^, accounting for 10.9% of total variance; ${\sigma }_{side}^{2}$ = 9.72 (95% CI: 0 to 40.27) N^2^, at 3.1% of total variance; and, ${\sigma }_{sess}^{2}$ = 4.83 (95% CI: 0 to 30.74) N^2^, 1.5% of total variance.

**Figure 3:**
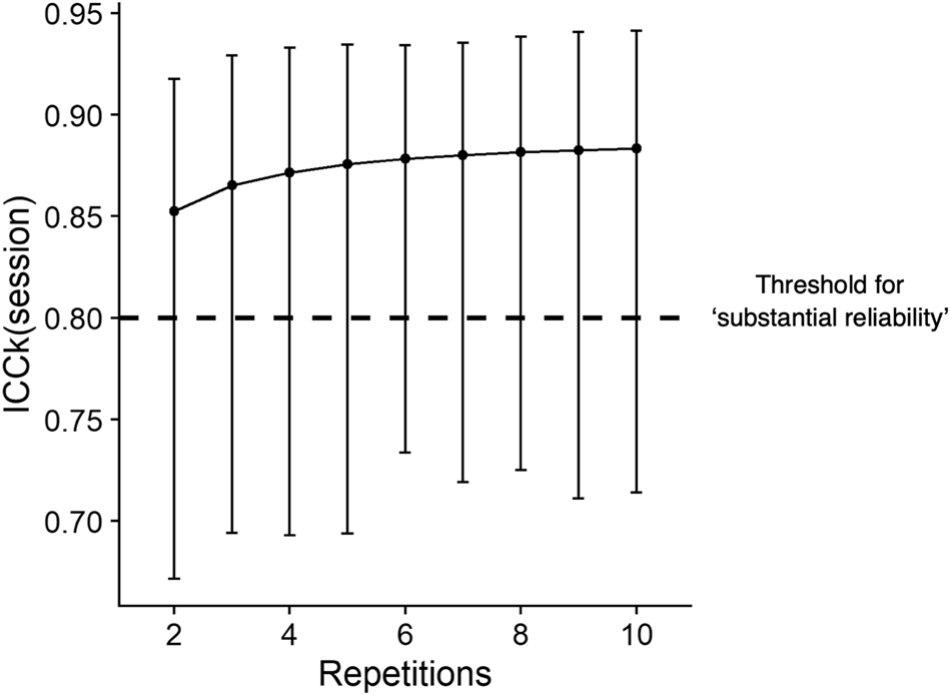
ICCk (session) values as a function of the number of repetitions.

The derived $ICC \left(sesssion\right), ICC \left(sesssion, reps\right)$, and $ICC \left(sesssion, side, reps\right)$, were calculated to be 0.88 (95% CI: 0.77 to 0.94), 0.73 (95% CI: 0.53 to 0.84), and 0.70 (95% CI: 0.49 to 0.81), respectively. When *L* was varied from two repetitions up to ten repetitions, the $ICCk\left(session\right)$ varied from 0.85 (95% CI: 0.67 to 0.92) to 0.88 (95% CI: 0.71 to 0.94), respectively (Fig. 3).

## Discussion

Studies investigating the reliability of PPT measurement typically incorporate multiple sources of variability^10^. To our knowledge, no studies have previously sought to identify and quantify the largest and smallest sources of PPT measurement variability as a proportion of total variance. The main finding of the present study was that the source of greatest variation was ${\sigma }_{subj}^{2}$ (70.1% of the total variance) while the source of least variation was ${\sigma }_{sess}^{2}$ (1.5% of total variance).

In a rare study that quantified individual sources of variation in PPT measurement^11^, the authors modelled sessions as a crossed-random effect. Two factors are crossed when every category of one factor co-occurs in the design with every category of the other; in other words, there is at least one observation in every combination of categories for both factors^17^. It therefore makes sense to treat sessions as crossed between subjects if all subjects’ sessions are synchronised (i.e. all first sessions for every subject occur at the same time, or at least on the same day, as do all second sessions, etc.). Given that this is impractical in any evaluation of PPT measurement, and impossible when using one rater, a more accurate statistical model would treat sessions as a *nested* within-subjects random effect. Hence, the present study modelled sessions as nested within subjects.

Using individual variance components to construct $ICC \left(session\right)$, we obtained ICC values comparable with those reported in the literature (i.e. between 0.85 to 0.98 in the lower back)^16^. However, previous investigators collapsed their data into only two sources of variation (i.e. ${\sigma }_{subj}^{2}$ and ${\sigma }_{sess}^{2}$)^16^, whereas we did not. In addition, because the present study comprised multiple sources of variation, our $ICC \left(session\right)$ was derived using Eq. (3). By contrast, $ICC \left(session\right)$ would be calculated using Eq. (1) in a study with only inter-session and inter-subject variability. However, if we had used Eq. (1) to calculate $ICC \left(session\right)$, inter-session reliability would have been calculated to be much higher at $\frac{222.28 }{222.28 +4.83}=$ 0.98. Hence, the present study provides evidence that when the methodology of a reliability study involves more than two sources of variability, collapsing data down to fewer sources of variability, to permit ICC calculation via traditional statistical software, may yield overoptimistic reliability estimates.

As a separate example, when evaluating reliability over different sessions, the items of a questionnaire do not change. This is certainly not the case with PPT measurement, where a single manual application of pressure cannot be perfectly replicated. Hence, a more comprehensive model of inter-session reliability $ICC \left(session, reps\right)$ accounts for the inescapable variability associated with different repetitions^17^. To our knowledge, no previous studies have accounted for inter-repetition variability when formulating inter-session ICC^16,24,29^. There is indirect evidence that inter-repetition variation may play a significant role when considering inter-session ICC. For example, higher inter-session reliability has been reported^4,24^ when the first PPT measurement was omitted from ICC calculations. Not surprisingly, $ICC \left(session, reps\right)$ yields a more conservative account of reliability than $ICC \left(session\right)$, which we consider to be clinically significant given that the interpretation of our value of $ICC \left(session\right)$ was that of ‘substantial’ reliability, and that for $ICC \left(session, reps\right)$ was of ‘moderate’ reliability.

In the present study, the $ICCk\left(session\right)$ improved from 0.85 when taking the average of two repetition to 0.88 when averaging over ten repetitions, respectively. Our $ICCk\left(session\right)$ results have indirect support from previous studies, which found averaging PPT values over multiple repetitions did not substantively change the interpretation of reliability results^4,16,24^. It is noteworthy that when previous studies have averaged PPT values over repetitions to derive inter-session ICC, they have been omitting the variance associated with repetitions, since there can be no variance of a single averaged value. This is in contrast to our formulation of $ICCk\left(session\right)$ in Eq. (6), where the variance associated with repetitions is not omitted, but instead reduces by a factor of $\frac{1}{KL}$. From Eq. (6), it can be deduced that the omission of the inter-repetition variability could explain why inter-session ICC increased to a greater extent (0.86 to 0.98 when averaging over three repetitions)^16^, than the present study. Evidently, incorporating all sources of measurement variability leads to a more conservative estimate for most ICC values.

Quantifying sources of variance for each measurement component not only enables the flexible calculation of different types of ICC to best reflect clinical or research practice, but the extracted variance components can also be used to derive measurements of agreement (e.g. standard error of measurement), although the latter was not the focus of the present study^30^. Given that the focus was on measurements of reliability, the main implication of our findings is that future reports of inter-session ICC should account for the variability associated with both multiple sessions and repetitions: our $ICC \left(session, reps\right)$.

The present study’s $ICC \left(session, side, reps\right)$ can be considered diametrically opposite to that of $ICCk\left(session\right)$. The former considers the correlation between PPT values of the same subject, but different sessions, sides, and repetitions, whilst the latter considers the correlation between averaged (across repetitions and sides) PPT values of the same subject in different sessions. Based on our $ICCk\left(sesssion\right)$ values, one clinically feasible strategy to optimise inter-session reliability would be to perform two repetitions per side of the lower back and take the average of all four values. This recommendation does not incur undue subject burden, clinician workload or resource cost, and is aligned with prior research recommending using the average of two repetitions^16,24^.

Given that ${\sigma }_{subj}^{2}$ was the source of greatest variance, one can speculate on how to manage the variability associated with testing different subjects. One study reported that male participants had 25% higher PPTs than female participants^31^, suggesting that ${\sigma }_{subj}^{2}$ could be reduced by including sex as an independent variable in the statistical model. In addition, another study reported that anxiety levels were negatively associated with PPT magnitude^32^. It is also possible that some of our participants could have greater experience undergoing PPT testing than others. Participants with greater PPT testing experience, may have heightened levels of self-efficacy, which may contribute to greater pain tolerance^33^. Future studies may benefit from quantifying participants’ prior experience with PPT testing and the presence of psychological factors. This information could be used within eligibility criteria, or as additional covariates in the statistical model, to potentially reduce high ${\sigma }_{subj}^{2}$.

This study is not without limitations. Firstly, we did not include multiple assessors, which would be necessary to provide an estimate of inter-session reliability when different clinicians measure PPT values on the same subject at different sessions. Secondly, this study investigated the reliability of PPT measurement in a cohort of healthy young adults, which may limit generalisability of the results to other age groups and to clinical populations. Lastly, we are aware that our study utilised relatively few testing sites. Future studies could include more testing sites so that variability between sites, within individuals, could be quantified within the statistical model.

## Conclusion

Inter-session-repetition ICC provides a more conservative estimate of reliability than inter-session ICC, with the magnitude of difference being clinically meaningful. Quantifying the amount of normal variability in repeated PPT measurement is of importance in research and clinical environments. The novelty of the present study is that by first quantifying the values of individual sources of variability, researchers and clinicians can construct relevant ICC values for clinically realistic situations, such as the present study’s inter-session-repetition ICC. Knowledge of individual sources of variability enables one to optimise future testing scenarios whilst balancing the cost of more laborious testing.

## References

[1] Scholten, P., Chekka, K. & Benzon, H. in *Essentials of Pain Medicine* (eds HT Benzon *et al.*) 27–38 (Elsevier, 2011).

[2] Fischer, A. A. Pressure algometry over normal muscles. Standard values, validity and reproducibility of pressure threshold. *Pain* **30**, 115–126, doi:10.1016/0304-3959(87)90089-3 (1987). 10.1016/0304-3959(87)90089-3 3614975

[3] Fechner, G. *Elements of Psychophysics.* (Rinehart and Winston, 1966).

[4] Tamara, E. L., Jan, H. H. & Lorenz, J. P. v. D. Experimental pressure-pain assessments: test-retest reliability, convergence and dimensionality. *Scand J Pain* **3**, 31–37, 10.1016/j.sjpain.2011.10.003 (2012). 10.1016/j.sjpain.2011.10.003 29913770

[5] Waller R, Straker L, O'Sullivan P, Sterling M, Smith A (2015). Reliability of pressure pain threshold testing in healthy pain free young adults. Scand. J. Pain.

[6] Nothnagel H (2017). How stable are quantitative sensory testing measurements over time? Report on 10-week reliability and agreement of results in healthy volunteers. J. Pain Res..

[7] Gomolka S (2019). Assessing endogenous pain inhibition: test-retest reliability of exercise-induced hypoalgesia in local and remote body parts after aerobic cycling. Pain Med..

[8] Tabatabaiee A, Takamjani IE, Sarrafzadeh J, Salehi R, Ahmadi M (2020). Pressure pain threshold in subjects with piriformis syndrome: test-retest, intrarater, and interrater reliability, and minimal detectible changes. Arch. Phys. Med. Rehabil..

[9] Knapstad MK (2018). Measuring pressure pain threshold in the cervical region of dizzy patients-The reliability of a pressure algometer. Physiother. Res. Int..

[10] Balaguier R, Madeleine P, Vuillerme N (2016). Intra-session absolute and relative reliability of pressure pain thresholds in the low back region of vine-workers: effect of the number of trials. BMC Musculoskelet. Disord..

[11] O'Neill S, O'Neill L (2015). Improving QST reliability—more raters, tests, or occasions? A multivariate generalizability study. J. Pain.

[12] Pryseley A (2009). Applying concepts of generalizability theory on data from experimental pain studies to investigate reliability. Basic Clin. Pharmacol. Toxicol..

[13] Shrout PE, Fleiss JL (1979). Intraclass correlations: uses in assessing rater reliability. Psychol. Bull..

[14] McGraw KO, Wong SP (1996). Forming inferences about some intraclass correlation coefficients. Psychol. Methods.

[15] Liljequist D, Elfving B, Skavberg Roaldsen K (2019). Intraclass correlation—a discussion and demonstration of basic features. PLoS ONE.

[16] Balaguier R, Madeleine P, Vuillerme N (2016). Is one trial sufficient to obtain excellent pressure pain threshold reliability in the low back of asymptomatic individuals? A test-retest study. PLoS ONE.

[17] Chia K, Sangeux M (2017). Quantifying sources of variability in gait analysis. Gait Posture.

[18] Koo TK, Li MY (2016). A guideline of selecting and reporting intraclass correlation coefficients for reliability research. J. Chiropr. Med..

[19] Zou GY (2012). Sample size formulas for estimating intraclass correlation coefficients with precision and assurance. Stat. Med..

[20] Evans DW, De Nunzio AM (2020). Controlled manual loading of body tissues: towards the next generation of pressure algometer. Chiropractic Manual Ther..

[21] Chesterton LS, Sim J, Wright CC, Foster NE (2007). Interrater reliability of algometry in measuring pressure pain thresholds in healthy humans, using multiple raters. Clin. J. Pain.

[22] Aspinall SL, Jacques A, Leboeuf-Yde C, Etherington SJ, Walker BF (2020). Pressure pain threshold and temporal summation in adults with episodic and persistent low back pain trajectories: a secondary analysis at baseline and after lumbar manipulation or sham. Chiropractic Manual Ther..

[23] Rolke R (2006). Quantitative sensory testing in the German Research Network on Neuropathic Pain (DFNS): standardized protocol and reference values. Pain.

[24] Nussbaum EL, Downes L (1998). Reliability of clinical pressure-pain algometric measurements obtained on consecutive days. Phys. Ther..

[25] Wasner GL, Brock JA (2008). Determinants of thermal pain thresholds in normal subjects. Clin. Neurophysiol..

[26] Kristensen JD, Svensson B, Gordh T (1992). The NMDA-receptor antagonist CPP abolishes neurogenic 'wind-up pain' after intrathecal administration in humans. Pain.

[27] Price DD, Mao J, Frenk H, Mayer DJ (1994). The N-methyl-D-aspartate receptor antagonist dextromethorphan selectively reduces temporal summation of second pain in man. Pain.

[28] Shrout PE (1998). Measurement reliability and agreement in psychiatry. Stat. Methods Med. Res..

[29] Walton DM (2011). Reliability, standard error, and minimum detectable change of clinical pressure pain threshold testing in people with and without acute neck pain. J. Orthop. Sports Phys. Ther..

[30] de Vet HCW, Terwee CB, Knol DL, Bouter LM (2006). When to use agreement versus reliability measures. J. Clin. Epidemiol..

[31] Melia M (2019). Pressure pain thresholds: subject factors and the meaning of peak pressures. Eur. J. Pain.

[32] Melia M (2015). Measuring mechanical pain: the refinement and standardization of pressure pain threshold measurements. Behav. Res. Methods.

[33] Bandura A, O'Leary A, Taylor CB, Gauthier J, Gossard D (1987). Perceived self-efficacy and pain control: opioid and nonopioid mechanisms. J. Pers. Soc. Psychol..

